# Chronic Constipation Ameliorated by Thoracic Spinal Cord Stimulation

**DOI:** 10.7759/cureus.57386

**Published:** 2024-04-01

**Authors:** Hiroshi Fujioka

**Affiliations:** 1 Department of Neurosurgery, Ohshima Hospital, Saga, JPN; 2 Department of Neurosurgery, Cognitive and Molecular Research Institute of Brain Diseases, Kurume University, Fukuoka, JPN

**Keywords:** gastrointestinal symptoms, neuromodulation, visceral pain, chronic constipation, spinal cord stimulation

## Abstract

A male in his mid-50s with a history of cerebral palsy was referred to the neurosurgical department for the management of chronic abdominal visceral pain after nine years of suffering. He had refractory constipation in his youth. Following a permanent colostomy for intestinal obstruction, visceral pain emerged over the right abdominal area, which became refractory to medication. Spinal cord stimulation (SCS) was performed with a pair of electrodes placed over the right mid-dorsal column between the T11-12 segments. Low-frequency stimuli with enough intensity to induce abdominal twitching reduced pain and relieved constipation for at least one year’s follow-up. As the effects were strong and persistent, our findings suggest a novel neuromodulation therapy for chronic constipation. However, clinicians should be aware of the potential risk of unwanted gastrointestinal symptoms when thoracic SCS is performed.

## Introduction

Chronic visceral pain is typically induced by gastrointestinal cancer, pancreatitis, or adhesions [1]. It significantly deteriorates patients’ quality of life and conventional therapies are limited [1]: analgesics and narcotics are often ineffective, and the benefits of neurolytic blocks are transient. Midline myelotomy and, less invasively, spinal cord stimulation (SCS) are promising neurosurgical options for the management of chronic, refractory visceral pain [2].

SCS is effective for visceral pain when the midline dorsal columns of thoracic levels are targeted [1-4]. Although transient and reversible, studies have reported SCS-associated unfavorable gastrointestinal symptoms such as nausea, diarrhea, or constipation [5-9]. While these symptoms suggest autonomic neuromodulation of gastrointestinal systems, therapeutic effects have been demonstrated only for the treatment of irritable bowel syndrome through T5-8 stimuli [10-12]. The present case illustrates the novel therapeutic potential of lower thoracic SCS for chronic constipation.

## Case presentation

A male in his mid-50s with a history of cerebral palsy (dyskinetic type) was referred to the neurosurgical department for the management of intractable abdominal pain after nine years of suffering. He had severe constipation in his youth. Following repeated episodes of intestinal obstruction due to ileus, he underwent permanent colostomy when he was 45 years old. Surgery was uneventful; however, pain over the right abdominal area was postoperatively recognized, which became refractory to any analgesics (nonsteroidal anti-inflammatory drugs, calcium channel α2-δ ligands, tricyclic antidepressant, selective serotonin reuptake inhibitor, and opioids). He abandoned taking analgesics because they had no effects.

**Figure 1:**
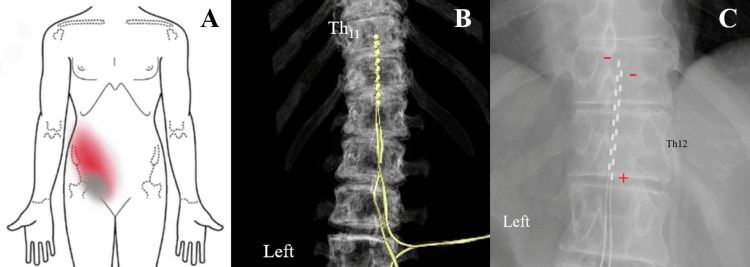
Visceral pain and SCS electrodes. Schematic drawing of the visceral pain over the right abdominal area (shown in blurred red) and the neuropathic pain over the pubic area (gray) (A). 3D-computed tomography (B) and X-ray (C) showing a pair of epidural SCS electrodes (+ indicates anode, - cathode) over the right mid-dorsal columns at the T11-12 vertebral levels. SCS: spinal cord stimulation.

Abdominal CT did not exhibit any abnormalities or lesions, including the site of colostomy. Thoraco-lumbar CT was unremarkable. Physical examination indicated a combination of visceral pain, which was diffusely recognized over the right lower abdomen, and neuropathic pain, which was localized on the right pubic area (Figure 1A). Using a numerical rating scale (NRS), the pain was consistently 8-10 points and was associated with severe sleep disturbance. Visceral pain was somewhat aggravated after eating but otherwise spontaneous. Allodynia was not noted. He was depressive in mood, which was sometimes associated with suicidal ideation.

Based on the favorable outcomes of SCS on refractory visceral pain [1-4], SCS was indicated. An SCS trial for one week was performed with a pair of epidural electrodes placed from the T8 down to the T12 vertebral level, wherein a moderate pain reduction was obtained at the T11-12 levels. Subsequently, a pair of SCS electrodes (eight channels in each lead; Medtronic Inc, Minneapolis, MN, USA) and an implantable pulse generator were implanted. The stimulation target was set over the right mid-dorsal columns at the T11-12 vertebral levels (Figure 1).

Continuous, low-frequency stimuli (5 Hz, pulse width 300 µsec) that were sufficient to induce abdominal twitching (3.0-4.0 V) achieved the maximum pain reduction. According to the patient, low-frequency stimuli with a “tap-tap” feeling produced the best therapeutic effect. The effect for pain was substantially alleviated when stimulation frequencies were set higher (> 40 Hz). The final adjustment of SCS reliably reduced visceral pain (5-6 points in NRS), although the effect was modest over the right pubic area (7-8 points in NRS).

No gastrointestinal changes were observed using the initial stimulation parameters; however, chronic constipation began to ameliorate a few weeks after the final adjustment of SCS, wherein a prominent acceleration of defecation and bowel gas was observed. Medication was not changed. The intestinal alterations were not associated with unwanted symptoms such as anorexia, nausea, or vomiting. Blood pressure was stable. The therapeutic effects for visceral pain and constipation persisted for at least about one year’s periodical follow-up, providing relief of pain (6 points in NRS) and sleep disturbance. Thereafter, follow-up was discontinued due to the author's relocation to another hospital.

## Discussion

A subset of patients with chronic constipation are refractory to any conventional therapies; thus, their therapeutic options are currently limited: digestive surgery is rarely indicated, and the effect of sacral neuromodulation is contradictory [13-14]. The present case demonstrated the therapeutic potential of SCS of the lower thoracic level, which was potent and persistent enough to meet the clinical demand.

As the patient suffered refractory constipation long before the emergence of abdominal pain, it is unlikely that reduction of pain was the direct cause of the amelioration of constipation. The involvement of medications is also unlikely since the patient did not take any analgesics, and medication was not changed throughout the SCS therapy.

Visceral pain is afferently transmitted from the abdominal viscera to the dorsal horn, and then to the postsynaptic midline dorsal column pathway [2, 15]. This midline spinal pathway has been regarded as a therapeutic target for intractable abdominal pain. Punctate midline myelotomy is performed through the selective disconnection of the pathway [16]. SCS for visceral pain likewise targets the midline dorsal columns at the mid- to lower thoracic levels (typically from T5-6 to T12 segments [1, 3, 4]), with the assumption of modulating the pathway [17]. In accordance with the reports by Kapural’s group [3, 4], the present case also demonstrated the effect of the SCS stimulation at the T11-12 segments.

It seems unlikely that the reduction of visceral pain through the above pathway was directly associated with the amelioration of constipation. It has been hypothesized that SCS exerts its therapeutic effect on visceral pain by modulating the sympathetic pathway that carries nociceptive information [3]. For the lower abdomen, the sympathetic pathway area includes the white ramus communicans and the lesser and/or least splanchnic nerve (T11-12 segments), which are preganglionic nerves originating from the lateral horn via the anterior root． 

The lesser and/or splanchnic nerves, which are sympathetic fibers that contain afferent pain information, are therapeutic targets in neurolytic blocks for visceral pain [15]. Transient diarrhea is often induced by the blocks via parasympathetic overactivation [11]. Since the stimulus intensity in the present case was strong enough to induce continuous abdominal twitching, we hypothesize that the electric current affected the sympathetic pathway on the ventral side, in addition to the midline dorsal column pathway.

Another line of evidence suggests the potential involvement of mechanical vibration of the abdomen. SCS in the present setting was a low-frequency stimulus with sufficient intensity to constantly induce mechanical twitches of the abdominal area. A recent clinical study reported the moderate therapeutic effects of orally ingesting a small, vibrating capsule that stimulates the intestine for chronic constipation [18]. Considering that this study used roughly the same vibrating frequency of 3-9 Hz as the present case (5 Hz), this suggests that abdominal movements via SCS contributed to the amelioration of constipation.

Sporadic cases documented that SCS is associated with gastrointestinal symptoms (Table 1). Thoracic SCS is associated with both diarrhea [6] and constipation [7, 9], which suggests that SCS changes sympathetic/parasympathetic balance according to stimulation parameters (frequency, intensity, polarity, and targeted area). To the best of our knowledge, there are no previous reports of gastrointestinal symptoms associated with lower thoracic SCS. Considering that lower thoracic levels are frequently used as a stimulation target for lumbar and lower limb pain, the potential risk of unwanted gastrointestinal symptoms is low. Further studies are needed to identify stimulation parameters and targets.

**Table 1:** SCS-associated gastrointestinal symptoms. SCS: spinal cord stimulation; Stim. sites: stimulation sites; NR: not reported; IBS: irritable bowel syndrome.

Article	References	Study design	Stim. sites	Outcome
Kemler et al.	[5]	single case	C4	Detrimental: ulcerative colitis relapsed
Thakker et al.	[6]	two cases	NR	Detrimental: nausea, flatulence, and diarrhea
Krames et al.	[10]	single case	T8-9	Beneficial: improved abdominal pain and diarrhea in IBS
La Grua	[7]	single case	T8	Detrimental: constipation, distension, and abdominal pain
Vorenkamp et al.	[8]	single case	T7-8	Detrimental: nausea
Rana et al.	[11]	single case	T8	Beneficial: improved pain and regulated bowel habits in IBS
Lind et al.	[12]	randomized cross-over study (n=9)	T5-8	Beneficial: improved pain in IBS
Manjunath et al.	[9]	single case	T8-9	Detrimental: severe constipation

## Conclusions

In conclusion, the present case demonstrated that lower thoracic SCS has therapeutic potential for chronic constipation when adequate stimulation parameters are chosen. Clinicians should also be aware of the potential risk that SCS for visceral pain can be associated with gastrointestinal symptoms.

Given the potent and long-lasting effects on constipation, clinical demand is considered high. However, it is unknown whether the present results in a cerebral palsy patient can be generalized. Accumulation of clinical data and experimental verification are necessary.

## References

[1] Khan YN, Raza SS, Khan EA (2005). Application of spinal cord stimulation for the treatment of abdominal visceral pain syndromes: case reports. Neuromodulation.

[2] Woodroffe RW, Pearson AC, Pearlman AM (2020). Spinal cord stimulation for visceral pain: present approaches and future strategies. Pain Med.

[3] Kapural L, Narouze SN, Janicki TI, Mekhail N (2006). Spinal cord stimulation is an effective treatment for the chronic intractable visceral pelvic pain. Pain Med.

[4] Kapural L, Nagem H, Tlucek H, Sessler DI (2010). Spinal cord stimulation for chronic visceral abdominal pain. Pain Med.

[5] Kemler MA, Gerald AM, van Kleef M (1999). Relapsing ulcerative colitis associated with spinal cord stimulation. Gastroenterology.

[6] Thakkar N, Connelly NR, Vieira P (2003). Gastrointestinal symptoms secondary to implanted spinal cord stimulators. Anesth Analg.

[7] La Grua M (2009). Rare side-effects during spinal cord stimulation: gastrointestinal symptoms. Neuromodulation.

[8] Vorenkamp KE, Baker NE (2010). Spontaneous resolution of nausea induced by spinal cord stimulation for failed back surgery syndrome. Neuromodulation.

[9] Manjunath A, Goel C, Baskaran AB, Kozel OA, Gibson W, Jones M, Rosenow JM (2023). Spinal cord stimulation-induced gastroparesis: A case report. Surg Neurol Int.

[10] Krames E, Mousad DG (2004). Spinal cord stimulation reverses pain and diarrheal episodes of irritable bowel syndrome: a case report. Neuromodulation.

[11] Rana MV, Knezevic NN (2013). Tripolar spinal cord stimulation for the treatment of abdominal pain associated with irritable bowel syndrome. Neuromodulation.

[12] Lind G, Winter J, Linderoth B, Hellström PM (2015). Therapeutic value of spinal cord stimulation in irritable bowel syndrome: a randomized crossover pilot study. Am J Physiol Regul Integr Comp Physiol.

[13] Pauwels N, Willemse C, Hellemans S (2021). The role of neuromodulation in chronic functional constipation: a systematic review. Acta Gastroenterol Belg.

[14] Bassotti G, Usai Satta P, Bellini M (2021). Chronic idiopathic constipation in adults: a review on current guidelines and emerging treatment options. Clin Exp Gastroenterol.

[15] Raj P (2001). Celiac plexus/splanchnic nerve blocks. Tech Region Anesth Pain Manage.

[16] Nauta HJ, Hewitt E, Westlund KN, Willis WD Jr (1997). Surgical interruption of a midline dorsal column visceral pain pathway. Case report and review of the literature. J Neurosurg.

[17] Krames ES, Foreman R (2007). Spinal cord stimulation modulates visceral nociception and hyperalgesia via the spinothalamic tracts and the postsynaptic dorsal column pathways: a literature review and hypothesis. Neuromodulation.

[18] Rao SS, Quigley EM, Chey WD, Sharma A, Lembo AJ (2023). Randomized placebo-controlled phase 3 trial of vibrating capsule for chronic constipation. Gastroenterology.

